# Loss of sphingosine 1-phosphate receptor 3 gene function impairs injury-induced stromal angiogenesis in mouse cornea

**DOI:** 10.1038/s41374-020-00505-1

**Published:** 2020-11-16

**Authors:** Shingo Yasuda, Takayoshi Sumioka, Hiroki Iwanishi, Yuka Okada, Masayasu Miyajima, Kana Ichikawa, Peter S. Reinach, Shizuya Saika

**Affiliations:** Department of Ophthalmology, Wakayama Medical University School of Medicine, 811-1 Kimiidera, Wakayama, 641-0012 Japan

**Keywords:** Transforming growth factor beta, Growth factor signalling, Angiogenesis

## Abstract

Sphingosine 1-phosphate (S1P) is a bioactive sphingolipid generated through sphingosine kinase1 (SPK1)-mediated phosphorylation of sphingosine. We show here that injury-induced S1P upregulation increases corneal neovascularization through stimulating S1PR3, a cognate receptor. since this response was suppressed in S1PR3-knockout mice. Furthermore, Cayman10444, a selective S1PR3 inhibitor, reduced this response in WT mice. Such reductions in neovascularization were associated with reduced vascular endothelial growth factor A (VEGF-A) mRNA expression levels in WT TKE2 corneal epithelial cells and macrophages treated with CAY10444 as well as macrophages isolated from S1PR3 KO mice. S1P increased tube-like vessel formation in human vascular endothelial cells (HUVEC) and human retinal microvascular endothelial cells (HRMECs) cells expressing S1PR3. In S1PR3 KO mice, TGFβ1-induced increases in αSMA gene expression levels were suppressed relative to those in the WT counterparts. In S1PR3 deficient macrophages, VEGF-A expression levels were lower than in WT macrophages. Transforming growth factor β1(TGFβ1) upregulated SPK1 expression levels in ocular fibroblasts and TKE2 corneal epithelial cells. CAY10444 blocked S1P-induced increases in VEGF-A mRNA expression levels in TKE2 corneal epithelial cells. Endogenous S1P signaling upregulated VEGF-A and VE-cadherin mRNA expression levels in HUVEC. Unlike in TKE2 cells, SIS3 failed to block TGFβ1-induced VEGF-A upregulation in ocular fibroblasts. Taken together, these results indicate that injury-induced TGFβ1 upregulation increases S1P generation through increases in SPK1 activity. The rise in S1P formation stimulates the S1PR3-linked signaling pathway, which in turn increases VEGF-A expression levels and angiogenesis in mouse corneas.

## Abstract (web summary)

Injury-induced transforming growth factor β1 increases sphingosine 1-phosphate (S1P) generation by upregulating in sphingosine kinase 1 activity. The high levels of S1P formation stimulate the S1PR3-linked signaling pathway, which in turn increases vascular endothelial growth factor-A expression levels and angiogenesis in mouse corneas.

## Introduction

Loss of corneal transparency following severe injury or infection may be due to maladaptive chronic neovascularization, inflammation and scarification. This undesirable outcome is induced by the activation of a host of different receptor-linked signaling pathways that are not self-limiting and result in corneal opacification. As there are no selective interventions to decrease any of these side effects in a clinical setting, novel therapeutics are needed to lessen declines in visual acuity caused by these sight disrupting responses to injury. Developing novel selective therapeutics remains a challenge since the pathophysiological mechanisms underlying the development of any of these sight compromising responses still require clarification. Initially following corneal injury, vascularization of a wound promotes healing through improving tissue oxygenation and access to nutrient support. Similarly, immune cell activation followed by infiltration into the wound removes cellular debris and reduces the likelihood of pathogenic infiltration into the corneal stroma. In addition, stromal extracellular matrix remodeling coupled with transdifferentiation of mesenchymal stem cells into myofibroblasts promote wound closure, but inflammation and neovascularization can instead induce corneal translucence if these responses are not self-limiting. As the current options to deal with any of these chronic responses are merely palliative, there remains a need to gain insight into the underlying molecular mechanisms and ligands that induce these responses through a myriad of different receptor- linked signaling pathways. Therefore, extensive effort has been dedicated to clarify the identity of, growth factors cognate receptors and linked signaling pathway axes whose expression levels are modulated in controlling the proliferation and infiltration of endothelial cell tube-like structures into the tissue as well as influx of activated immune cells and myofibroblast transdifferentiation. The delineation of the roles of TGFβ and VEGF to the wound healing process has aided efforts to identity targets for preventing this response from becoming dysregulated and preventing restoration of tissue transparency [1–3]. Nevertheless, developing agents that selectively interact with these sites remains a challenge. S1P is a bioactive lipid which serves as a multi-functional signaling mediator of cell growth regulation in vascular and immune systems [4–7]. Hla reported that S1P is a ligand of vascular endothelial differentiation gene 1 (EDG-1), a vascular differentiation-related receptor [8]. EDG-1 is currently designated as the S1P receptor 1 isoform (S1PR1). There are four other related members of this SP receptor family referred to as S1PR2-5 [9–11] in different tissues. Each of these different S1PR isoforms exhibits a specific expression pattern in tissues. S1PR1-3 isoforms are widely expressed in many tissues, while S1PR4 and S1PR5 are mainly expressed only in bone marrow- related cell linages and nervous systems [12]. S1PR1 and S1PR3 are both expressed in vascular endothelial cells [13]. Cell culture experiments suggest that the S1P/S1PR3-induced signaling stimulates neovascularization through a complex chain of events [14–16]. Sphingosine is generated from ceramide and is phosphorylated by a sphingosine kinase 1 (SPK1) to yield S1P. S1P expression levels are established as a balance between SPK1 generation of S1P and its dephosphorylation by phosphatases as well as lyases activities. Even though it is known that VEGF modulates S1P expression levels in some other tissues, such an interaction has not been described in the cornea [17]. Similarly, even though there are known interactions between S1P mediated signaling and the fibrotic responses induced by interactions with TGFβ, there are no reports describing crosstalk between TGFβ linked and S1PR3-linked signaling pathways in the cornea [18, 19]. We describe here the involvement of the S1P/S1PR3signaling pathway axis in mediating neovascularization during corneal wound healing. This response induced by cauterization was compared in a S1PR3-null (KO) mouse with that in its WT counterpart. As loss of S1PR3 attenuated injury-induced corneal neovascularization, the effects of S1P, TGFβ and VEGF were also determined on HUVEC, human retinal microvascular endothelial cells (HRMECs), murine fibroblasts and TKE2 corneal epithelial cells to characterize how S1PR3 activation induces neovascularization. The results show that crosstalk among cell signaling mediators in the pathways linked to VEGF and TGFβ cognate receptors with those in the S1P/S1PR3 cell signaling pathway axis modulate corneal injury-induced neovascularization in mice. The identity of such interactions provides additional targets whose modulation by selective agents may ultimately improve management of the corneal wound healing response induced by a penetrating injury to this tissue.

## Materials and methods

Each experimental protocol was approved by the DNA Recombination Experiment Committee and the Animal Care and Use Committee of Wakayama Medical University, and performed in accordance with the Association for Research in Vision and Ophthalmology Statement for the Use of Animals in Ophthalmic and Vision Research.

### Immunohistochemistry of S1PR3 Expression

The protein expression pattern of S1PR3 in a C57/Bl6 (WT) mouse cornea was examined with an anti-S1PR3 antibody (Santa Cruz Biotechnology, Santa Cruz, CA, USA). Immunolabeling was evaluated based on the intensity of the 3,3’-diaminobenzidine color reaction. To evaluate S1PR3 expression flat-mounted epithelial debrided corneas were immunostained with an anti- S1PR3 antibody at day 7 post-cauterization (Santa Cruz) [13]. An anti-CD31 antibody (Santa Cruz) probed for stromal endothelial cell infiltration. Epithelial debridement was performed to obtain a more distinct CD31 and S1PR3 labeling of endothelial cells.

### Neovascularization in corneal stroma

S1PR3 KO mice obtained from MMRRC, UC, Davis CA, USA (*n* = 69) and WT mice (*n* = 70) assessed the role of S1P-induced signaling in mediating neovascularization. Light microscopic examination compared tissue organization and structure between these two genotypes. The center of the cornea of one eye of each mouse was cauterized by using a disposable coagulator (Beaver- Visitec Intenational, Waltham, MA, USA) in order to induce vessel extension from the limbus towards the corneal center [20, 21]. After different time intervals, mice were sacrificed and either paraffin-sectioning, or cryosectioning was performed of the cauterized eyes in preparation for performing immunohistochemistry. Alternatively, flat-mounts were used for this purpose.

### Histology and immunohistochemistry

At day 7 post-cauterization, the mice were sacrificed and flat-mount corneal specimens were prepared (WT, *n* = 5; KO, *n* = 5). Uninjured WT (*n* = 5) corneas were also used. CD31- immunohistochmeistry was performed on each sample to evaluate limbal vessel inward extension. The length was measured of CD31-labeled endothelial cell protrusion from the limbus towards the center in cryosection samples. KO mice (*n* = 44) and WT mice (*n* = 40). Sixteen, 15, and 13 KO corneas and 16, 14, and 10 WT corneas were processed for cryosectioning on days 3, 7, 14, respectively. CD31 immunohistochemistry was performed on each sample and the distance was measured between the leading tip of CD31-labeled vessels in the stroma and the anterior chamber angle. Mean values of their length on both sides of the cornea were used to evaluate the extent of neovascularization. The Mann–Whitney U test was used for data analysis. Five, 5, 5, and 5 KO corneas and 5, 5 5, and 5 WT corneas were processed for 5 μm thick paraffin-sections on days 3, 7, and 14, of cauterized and uninjured tissue, respectively. Samples were processed for immunohistochemistry to evaluate the expression level of VEGF-A, (R&D systems, Minneapolis, MN, USA) and α- smooth muscle actin (αSMA) (Thermo Fisher Scientific, Waltham, MA, USA).

### mRNA gene expression levels in in vivo samples

Uninjured WT (*n* = 52) and KO (*n* = 52) mice 3 days after cauterization were sacrificed. One eye of each mouse was used for RNA extraction. Tissue was treated with tissue lysis buffer (Sigma- Aldrich, St. Louis, MO, USA) and RNA was extracted as previously reported [19], Four corneas were pooled to constitute a single sample. Taqman real-time qRT-PCR was performed to semi- quantify the expression level of each target gene. Primers used are shown in Table 1. Data were analyzed by employing Student’s *t* test or Welch’s *t*-test.

**Table 1:** Primer sets used (Applied Biotechnology, Inc.).

VEGF-A	Mm01281447_m1, Hs00900055_m1
VEGF-B	Mm00442102_m1
TGFβ1	Mm03024053_m1
MPO	Mm00447886_m1
F4/80	Mm00802529_m1
αSMA	Mm01204962_gH
Sphingosine kinase 1	Mm00448841_g1
VE-cadherin(CDH5)	Hs00901465_m1

*VEGF-A* Vascular Endothelial Growth Factor-A, *VEGF-B* Vascular Endothelial Growth Factor-B, *TGFβ1* Transforming Growth Factor β1, *MPO* Myeloperoxidase, *αSMA* α-Smooth Muscle Actin

### Effect of TGFβ1 on sphingosine kinase1 expression in vitro

Sphingosine kinase 1 (SPK1) generates S1P from sphingosine, which induces a host of different responses through selectively interacting with one or more different cognate S1PR1-5 subtypes in different cell types [9–11]. The effects were determined of exogenous TGFβ1 (1 ng/ml, R&S System) on the expression level of SPK1 in either the TKE2 mouse corneal epithelial cell line (ECACC, Salisbury, UK, originally established by Kawakita et al. [22]) or cultured ocular fibroblasts, which were isolated from an eye on postnatal day 1 WT mouse [23, 24]. Both cell types were grown to confluence in a 60 mm culture dish. Then the cells were further incubated in a serum-free medium for 16 h. The cultures were then grouped in four different culture conditions and incubated for 24 h; (a) control serum-free medium; (b) TGFβ1 (1.0 ng/ml); (c) SIS3 (1 nM, Sigma-Aldrich); (d) TGFβ1 and SIS3 [25]. Ten dishes were prepared for each culture condition. RNA was then extracted from each culture processed for TaqMan real-time qRT-PCR of SPK1 mRNA [22–24]. Data were analyzed by employing Mann–Whitney U test.

### S1P-S1PR3 signaling modulation of VEGF-A expression

The effects were compared of exogenous S1P and/or a S1PR3 antagonist (CAY10444) on SPK1 expression in TKE2 corneal epithelial cells or ocular fibroblasts. Both of these cell types express S1PR3 as detected by immunohistochemistry. They were grown to confluence in a 60 mm culture dish. Then the cells were incubated in a serum-free medium for another 16 h. Cells were cultured under the following conditions for another 24 h under one of the following four different conditions: (a) S1P (200 nM) (Cayman Chemical, Ann Arbor, MI, USA); (b) CAY10444 (a selective S1PR3 antagonist, 100 μM, (Cayman Chemical); (c) or both S1P and CAY10444 at the aforementioned concentrations. (d) serum- free culture served as the control. Ten dishes were prepared for each culture condition. RNA was extracted and processed for real-time qRT-PCR for VEGF-A. Mann–Whitney U test was used for data analysis.

### Expression of VEGF-A in WT and S1PR3KO macrophages

Macrophages were obtained from WT and S1PR3 KO mice [24]. In brief, macrophages were harvested from the peritoneal cavity of each mouse by irrigating them with 10% serum-plus medium 4 days after i.p. injection of sterilized 5% oyster glycogen (1.0 ml, Sigma-Aldrich). Cells were cultured in 60 mm-culture dishes for 6 h. Five dishes were prepared each containing either WT or KO macrophages Non-adherent cells were washed out with phosphate-buffered saline (PBS). RNA was extracted and processed for TaqMan real-time qRT-PCR for VEGF-A. Data were statistically analyzed by employing Student’s *t* test or Welch’s *t*-test.

#### Expression of VEGF-A and VE-cadherin in human retinal microvascular endothelial cells (HRMECs) and human umbilical vein endothelial cells (HUVECs)

HRMECs (Cell Systems, Kirkland, WA, USA) or HUVECs (Kurabo, Osaka, Japan) were defrosted in either 5% serum-plus CS-C medium (Cell Systems) or 5% serum-plus CS-C medium (Kurabo) in a T-75 flask. The flasks were placed in a humidified CO_2_ incubator at 37 °C and cultured until they reached 80% confluence. Immunohistochemistry documented S1PR3 expression in both cell lines. The cells were cultured in 60 mm-culture dishes until they reached confluence and maintained under this condition for another 16 h in serum-free medium. In the serum-free medium, the cells were then exposed to either S1P (200 nM) or combined with CAY10444 (10 μM) for 24 h. Ten dishes were prepared for each culture condition. Aforementioned S1P and CAY10444 concentrations were chosen based on [26]. TaqMan real- time qRT-PCR RNA evaluated VEGF-A and VE-cadherin expression levels in RNA extracts of either HRMECs or HUVECs. The Mann–Whitney U test was used to analyze significance. Immunofluorescent cytochemistry was used to evaluate VE-cadherin protein expression levels in confluent HUVECs with an anti-VE-cadherin antibody (Abcam, Cambridge, UK) cultured in the wells of a chamber slide under the same condition as described above.

### Vessel-like structure formation in HRMECs

HRMECs (1 × 106 cells/25 cm^2^) were grown in a T75 flask for 24 h until they reached 80% confluence. The cells were rinsed in PBS and incubated for another 30 min in the medium supplemented with 2 μM calcein-AM. The cells were then trypsinized and centrifuged at 1000 x *g* for 5 min. Cultrex® reduced growth factor basement membrane matrix type 2 expression (RGF BME, 40 μL, Trevigen, Gaithersburg, MD, USA) was placed in a fluorescein-observation 96- well plate and placed for 30 min in the tissue culture incubator. The cells (3 × 104/100 μL) were then seeded in each well of the plate. Six experimental culture conditions were prepared containing combinations of three different reagents; They included S1P, CAY10444) and fetal bovine serum (FBS). Culture conditions were as follows; (1) control culture, (2) 200 nM S1P, (3) 200 nM S1P combined with 1 μM CAY10444, (4) 200 nM S1P combined with 10 μM CAY10444, (5) 200 nM S1P combined with 100 μM CAY10444, and (6) 10% FBS. After 10 h of incubation, the fluorescein stained culture was photographed under a microscope. Pictures were examined by using WinROOF (MITANI Corp, Japan) and the cell free area in a cell-free honeycomb structure was determined. Mann–Whitney U test was used to determine statistical significance.

## Results

### Expression pattern of S1PR3

**Fig. 1:**
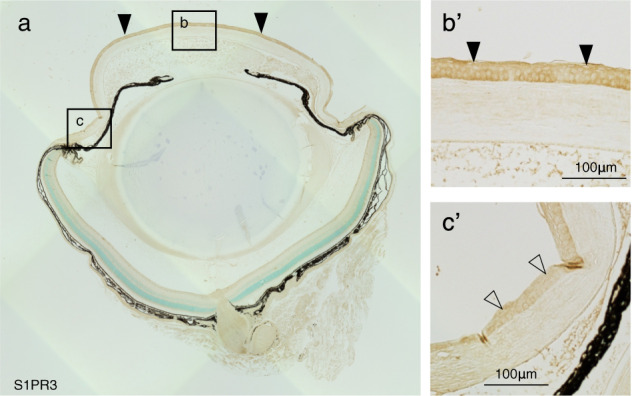
Expression pattern of S1PR3. **a** Immunohistochemistry evaluation was performed of S1PR3 expression levels based on intensity of 3,3’-daminobenzidine color development. S1PR3 is markedly expressed in each layer of the corneal epithelium (black arrowheads), while limbal epithelium (**c’** open arrowheads) stains faintly for S1PR3. Frames **b’** and **c’** are higher magnification images of areas enclosed by boxes with white colored borders. **b** and **c** in Frame **a**, respectively. Immunolabeling in the corneal stroma is very faint. Scale Bar, 100 μm, Epi, corneal epithelium.

**Fig. 2:**
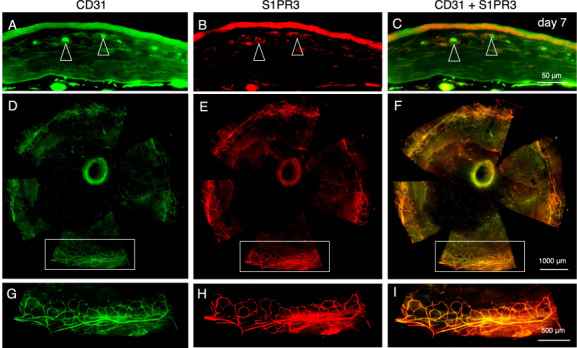
Expression pattern of S1PR3. Immunohistochemistry of a cryosection shows that the epithelium and the CD31-positive endothelial cells express S1PR3 (open arrowheads) at day 7 post-cauterization. To confirm the co-localization of S1PR3 and CD31 we performed immunostaining in an epithelium-debrided flat-mounted specimen. Cauterization-induced CD31-positive new vessels extending from the limbus that coexpresed S1PR3 at day 7. Frames **g**, **h**, and **i** are higher magnification images of enclosed by boxes with white colored borders in each of the Frames **d**, **e**, **f**, respectively. Scale Bar, 50, 1000, 500 μm.

To determine if S1P bioactivity is attributable to its interaction with S1PR3, we probed for S1PR3 expression since it is one of the three S1PR isotypes mediating eyelid closure during development in mice [27]. S1PR3 expression was evident in each layer of the corneal epithelium while its expression was faint in the limbal epithelium (Fig. 1). On day 7 post cauterization, infiltration of endothelial cells bearing S1PR3 was evident based on CD31 and S1PR3 co-immunostaining (Fig. 2a–c). An epithelium-debrided flat- mounted specimen was used to improve their staining pattern. Indeed, CD31-positive immunostained vessels co-labeled with S1PR3 were evident extending from the limbus at day 7 (Fig. 2d–i). Therefore, infiltration of CD-31 immunopositive cells into the stroma is associated with S1PR3 upregulation.

### Effect of the loss of S1PR3 function on stromal neovascularization

**Fig. 3:**
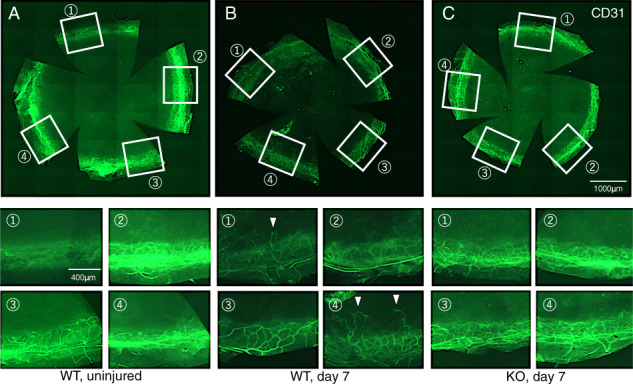
Limbal neovascularization in a S1PR3-null mouse. Flat-mounted specimen of the treated corneas suggests that S1PR3 gene deletion suppressed development of neovascularization as compared with a wild-type (WT) mouse at day 7. **a** In an uninjured WT mouse cornea, CD31-labeled vessels do not penetrate into the cornea interior. **b** In a WT cornea at day 7 post-cauterization CD31-labeled vessels do not extend into the cornea sprouts (arrowheads). **c** Such sprouts of the vessels are not observed in a KO post-cauterization cornea. Circled numbers 1–4 in the bottom two rows provide higher magnification images of the aforementioned described boxed in areas shown in each of Frames **a**, **b** and **c**. Bar, 1000 μm.

**Fig. 4:**
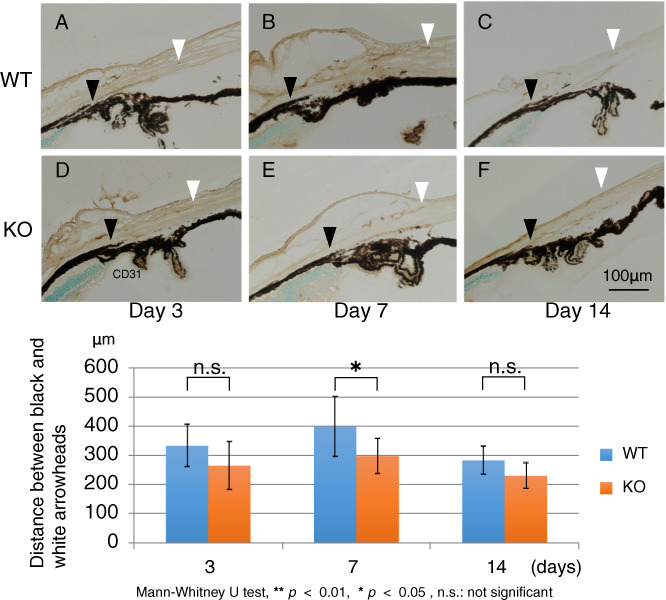
Effect of the loss of S1PR3 on corneal neovascularization. The length of new vessels in the cornea at each timepoint, was evaluated based on the distance between the anterior chamber angle and the tip of the neovascularization in the corneal stroma identified with CD31 immunolabeling in cryosections. **a** Distance between limbus (black arrowhead) and the tip of a new vessel (white arrowhead) represents the length of the new vessel in each frame. Frames A, B and C, or those of D, E, and F indicate wild-type (WT) or S1PR3- null (KO) cornea, respectively. Frames A and D, B and E, or C and F, indicate samples of day, 3, 7, or 14 post-cauterization, respectively. Scale Bar, 1000 μm. **b** The length of the new vessel was significantly shorter in a KO mouse as compared with that in a WT mouse at day 7, but not at days 3 and 14.

Stromal neovascularization was markedly less in S1PR3 KO than in their WT counterpart on day 7 in flat-mounted specimens (Fig. 3). CD31 immunostaining identified the leading edge of endothelial cell extension I into the stroma at each time point based on measurements of the distance between anterior chamber angle and the tube tip in cryosections. Their lengths were significantly shorter in a KO mouse as compared with that in a WT mouse at day 7, but not at days 3 and 14 (Fig. 4). Therefore, S1P-induced increases in neovascularization are dependent on S1PR3 expression.

### Gene expression of wound healing-related mediators in a cauterized cornea

**Fig. 5:**
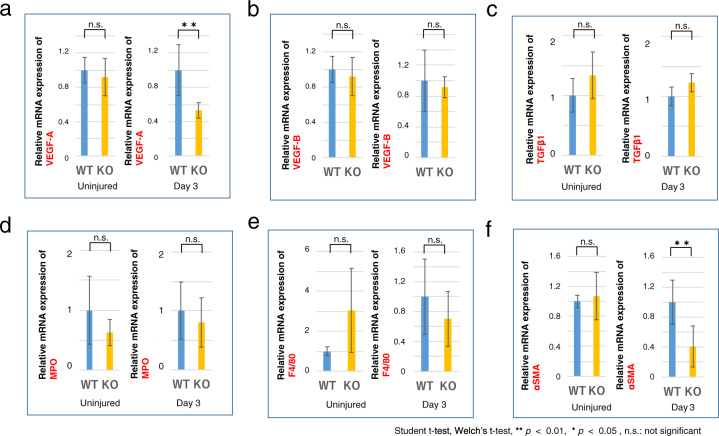
mRNA expression of wound healing-related mediators in a cauterized cornea. Expression level of vascular endothelial cell growth factor (VEGF)-A (**a**) was suppressed by the loss of S1PR3 at day 3, while that of VEGF-B (**b**) and transforming growth factor β1 (TGFβ1, **c**) were unaffected, in a cauterized cornea at day 3. mRNA expression level of wound healing- related cellular mediator was then examined. Expression level of myeloperoxidese (MPO, neutrophil marker, **d**) and F4/80 (macrophage marker, **e**) were unaffected by the loss of S1PR3 at day 3 in both cauterized and uninjured corneas as well as in uninjured samples. Expression of α-smooth muscle actin (αSMA, myofibroblast marker, **f**) was suppressed in an S1PR3-null (KO) tissue as compared with a wild- type (WT) one at day 3.

The role of S1PR3 in mediating S1P control of VEGF-A and V-EGF-B expression was evaluated by determining the effect of loss of S1PR3 function on their gene expression levels. In a cauterized cornea at day 3. VEGF-A RNA expression level was lower in the S1PR3 KO than in their WT counterpart. (Fig. 5a) In contrast, the expression levels of both VEGF-B (Fig. 5b) and TGFβ1 (Fig. 5c) were unaffected, Cauterization-induced MPO-positive neutrophil (Fig. 5d) and macrophages (F4/80-positive, Fig. 5e) infiltration was unaltered by the loss of S1PR3 function at day 3. based on no significant difference in their expression levels between the S1PR3 KO and the WT counterpart. However, myofibroblast transdifferentiation was suppressed based on a lower αSMA biomarker expression level in S1PR3 KO stroma than in a WT counterpart at day 3 (Fig. 5f).

### Immunohistochemical evaluation of changes in wound healing-related mediators

**Fig. 6:**
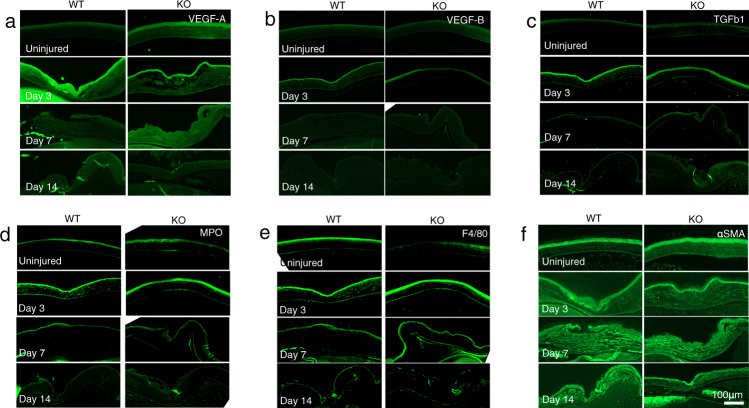
Immunohistochemical detection of wound healing-related mediators. The epithelium is the major source of VEGF-A was and its expression declined during the healing interval after day 3 (**a**). VEGF-A staining intensity is less marked in a S1PR3-null (KO) epithelium as compared with a wild-type (WT) tissue at day 3 (**a**). Protein expression of VEGF-B (**b**) and TGFβ1 (**c**) are similar in WT and KO tissues. Infiltration of myeloperoxidese (MPO)-labeled neutrophil is more prominent in a WT stroma (asterisk) as compared with a KO tissue (star) at day 3 (**d**). F4/80-positive macrophage population (arrows) was unaffected by the loss of S1PR3 during intervals examined up to day 14 (**e**). Immunohistochemistry showed protein expression of αSMA(myofibroblast marker) was overall less intense in KO tissues as compared with WT tissues throughout the healing interval (**f**). Bar, 100 μm, Epi, corneal epithelium.

Subsequent to wounding, the VEGF-A immunocytochemical staining was more pronounced in the epithelium than in the stroma, but 3 days after injury its expression level decreased. (Fig. 6a). At day 3, the VEGF-A staining intensity was less marked in the epithelial layer of S1PR3 KO mice compared with its level in the WT tissue (Fig. 6a). On the other hand, protein expression levels of VEGF-B (Fig. 6b) and TGFβ1 (Fig. 6c) appeared very similar to one another between the WT and KO tissues. Infiltration of MPO-labeled neutrophil was more prominent in the WT stroma (asterisk) as compared with that in the KO tissue (star) at day 3 (Fig. 6d). F4/80-positive immune-stained macrophage content was unaffected by the loss of S1PR3 function during intervals lasting up to day 14 (Fig. 6e). αSMA staining of myofibroblasts was overall less apparent in KO tissues as compared with WT tissues throughout the healing interval (Fig. 6f). The selective inhibitory effects of the loss of S1PR3function on VEGF-A and myofibroblast marker expression levels suggest that S1P contributes to mediating both injury-induced neovascularization and fibrosis.

### Differential Roles of TGFβ1/Smad3 signaling in controlling SPK1 mRNA gene expression levels

**Fig. 7:**
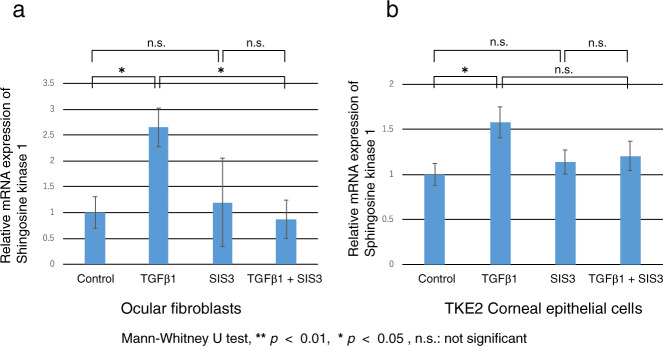
Roles of TGFβ1/Smad3 signaling in mediating expression of sphingosine kinase 1 mRNA in TKE2 corneal epithelial cell line and primary ocular fibroblasts. **a**, **b** Adding TGFβ1 to the medium upregulated sphingosine kinase 1 (SPK1) mRNA in both theTKE2 corneal epithelial cell line and primary ocular fibroblasts. Such upregulation was eliminated in the presence of SIS3, Smad3 inhibitor, in fibroblasts (**a**), but not in TKE2 corneal epithelial cell line (**b**).

The TGFβ1/Smad3 signaling pathway axis is one of the major cascades mediating control of wound healing in an injured cornea. To clarify if there is an interrelationship between the S1P/SPR3 signaling pathway axis and the TGFβ1/Smad3 signaling pathway, we examined the effects of either exogenous TGFβ1 and/or Smad3 signaling on SPK1 expression. SIS3 is a potent inhibitor of both Smad3 and SPK1 which is the major kinase that phosphorylates sphingosine to generate S1P in the TKE2 cell line or ocular fibroblasts (Fig. 7). Adding TGFβ1 to the medium upregulated SPK1 mRNA expression levels in both of these different cell types. Subsequent addition of SIS3, completely blocked the aforementioned SPK1 mRNA upregulation in the ocular fibroblasts. Interestingly, S1S3 had no effect on TGFβ1-induced SPK1 upregulation in the TKE2 cells (Fig. 7). These disparate findings indicate that (1) TGFβ1 modulates corneal wound healing through different pathways that phosphorylate Smad3 [28] and induce S1P biosynthesis. Secondly, SPK1 expression is only a modulator of wound healing responses in corneal fibroblasts, but not in TKE2 cells, respectively.

### Differential Roles of S1PR3 signaling in mediating control of VEGF-A mRNA expression levels

**Fig. 8:**
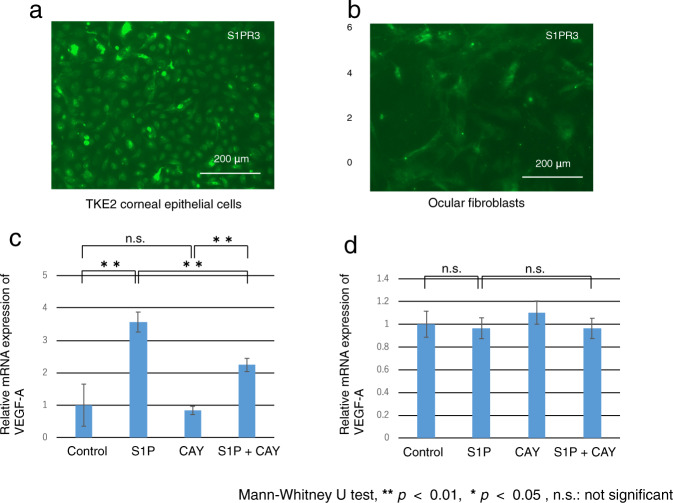
Roles of S1PR3 signaling in mediating the expression of VEGF-A mRNA in TKE2 corneal epithelial cell line and primary ocular fibroblasts. Both TKE2 corneal epithelial cells (**a**) and primary ocular fibroblasts (**b**) express S1PR3. Scale Bar, 200 μm. Administration of S1P upregulated VEGF-A, that declined following addition of an S1PR3 antagonist, CAY10444 (**c**). On the other hand, administration of S1P and/or CAY10444 did not affect the expression level of VEGF-A in ocular fibroblasts (**d**).

To determine if stimulation of the S1P/S1PR3 signaling pathway axis has effects on VEGF-A expression in the TKE2 cell line that are different than in ocular fibroblasts, we probed for S1PR3 expression in both types of tissues. Only the TKE2 cells immunostained with the anti S1PR3 antibody (Fig. 8a, b). In these cells, exogenous S1P upregulated VEGF-A mRNA expression levels whereas the S1PR3 antagonist, CAY10444 suppressed this effect. On the other hand, administration of S1P and/or CAY10444 did not affect the expression level of VEGF-A in ocular fibroblasts (Fig. 8c, d). Therefore, S1PR3 is only involved in mediating S1P control of VEGF expression levels in TKE2 cells.

### Expression of VEGF-A mRNA in WT and KO macrophages

**Fig. 9:**
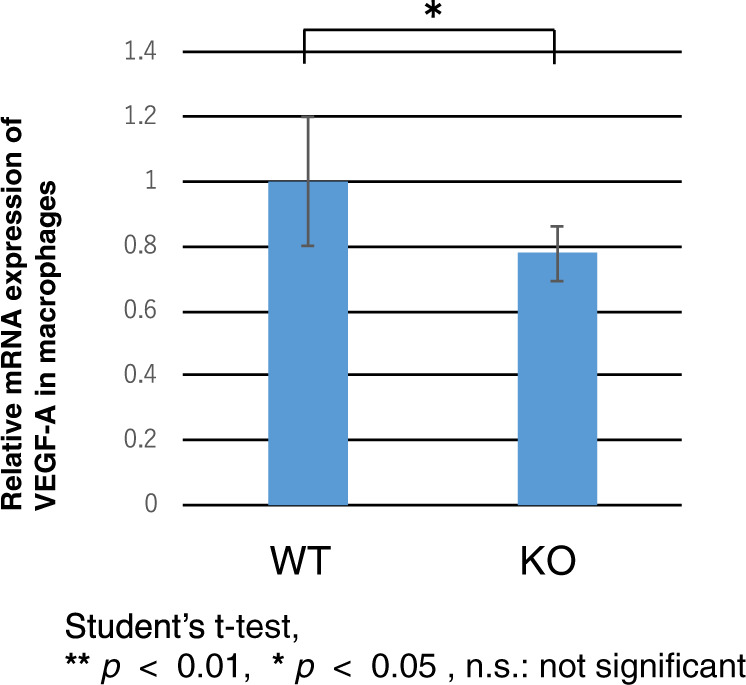
Expression of VEGF-A mRNA in wild- type (WT) and S1PR3-null (KO) macrophages. Expression level of VEGF-A mRNA in KO macrophages was significantly less as compared with that in WT macrophages.

The involvement was evaluated of the S1P/ SPR3 signaling pathway axis in controlling the VEGF-A gene expression by comparing the effects of S1P on VEGF-A gene expression levels in WT macrophages with those isolated from S1PR3 KO mice. Four days after injecting medium containing oyster glycogen into the abdominal cavity, the medium was retrieved and cultured for an additional 6 h. After 6 h, dish adherent macrophages were harvested and qRT-PCR was performed to evaluate VEGF mRNA expression levels. The results shown in Fig. 9 indicate that this signaling pathway contributes to the control of VEGFA gene expression since its expression level in S1PR3 KO macrophages was significantly less than in WT macrophages.

### Differential roles of S1PR3 signaling in mediating control of VEGF-A and VE-cadherin mRNA expression levels in HRMECs and HUVECs

**Fig. 10:**
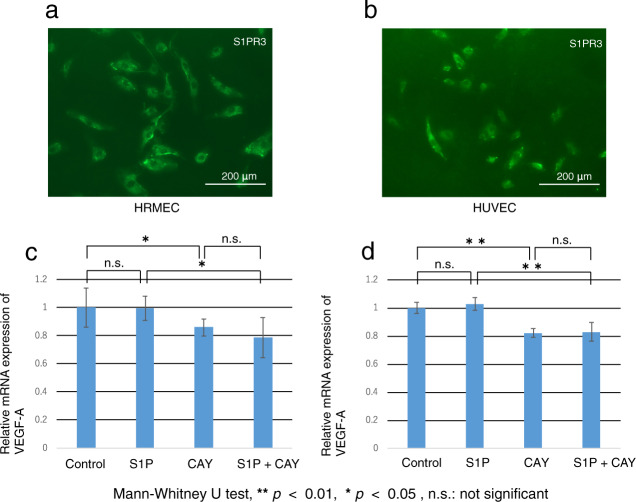
Roles of S1PR3 signaling in mediating the expression of VEGF-A mRNA in human retinal microvascular endothelial cells (HRMEC) and human umbilical vein endothelial cells (HUVEC). Both HRMEC (**a**) and HUVEC (**b**) were immunohistochemically labeled for S1PR3. Scale Bar, 200 μm. The role of the S1P/S1PR3 signaling pathway axis in mediating VEGF-A expression was analyzed based on the individual effects of either of an S1PR3 antagonist, CAY10444 on the expression of VEGF-A gene expression levels in these cell types. Administration of S1P to the medium did not alter the expression level of VEGF-A in both cell types. However, adding CAY10444 alone to the medium downregulated VEGF-A expression and its supplementation in a S1P containing medium blocked S1P’s induced VEGF-A upregulation in both cell types (**c**, **d**).

**Fig. 11:**
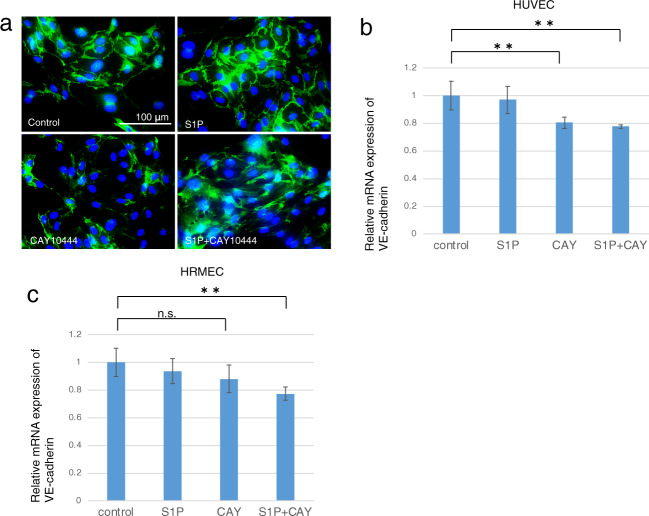
Roles of S1PR3 signaling in mediating the expression of VE-cadherin mRNA in HRMEC and HUVEC. **a** Immunocytochemistry documented protein expression of VE-cadherin. It was suppressed by adding CAY10444 to HUVECs culture. Bar, 100 μm. Adding S1P to the culture medium did not alter the expression level of VE-cadherin mRNA in both HUVECs (**b**) and HRMECs (**c**). On the other hand, supplementation of CAY10444 to the medium suppressed VE-cadherin expression irrespective of the presence or absence of S1P in cultures of both cell types.

The results shown Fig. 10a, b document S1PR3 subtype expression in both human retinal microvascular endothelial cells (HRMEC) and human umbilical vein endothelial cells (HUVECs). The roles were evaluated of S1PR3 signaling in mediating control of VEGFA gene expression levels and neovascularization in these two different cell types. To make this assessment, we determined if either the ligand S1P or a selective S1PR3 antagonist, CAY10444, affects their VEGFA mRNA expression levels. Interestingly, exogenous S1P did not alter the expression level of VEGF-A whereas CAY10444 downregulated VEGF-A expression and its supplementation in the S1P-containing medium eliminated S1P’s upregulation of VEGF-A in both cell types (Fig. 10c, d). These results suggest that only variations in endogenous S1P levels modulate VEGF-A gene expression levels while exogenous S1P supplementation do not modulate this response, One possibility for this discrepancy is that VEGF-A gene expression levels are maximally stimulated by endogenous S1P. Therefore exogenous S1P supplementation failed to augment the VEGF gene expression level. VE-cadherin is a cadherin family member that chiefly controls the opening and closing of the endothelial tight junctional barrier. This modulation affects transcellular solute penetrance from the vasculature into the extracellular space [29]. VEGF-A activation upregulates VE-cadherin expression [30, 31] and it is essential for inducing angiogenesis Using immunocytochemistry, the results shown in Fig. 11a reveal that inhibiting S1PR3 with CAY10444 suppressed VE-cadherin protein expression in HUVEC cultures (Fig. 11a). S1P and/or CAY10444 supplementation had opposing effects on the mRNA expression levels of VE-cadherin and on VEGF-A expression levels that were similar with one another in HUVEC and HRMEC (Fig. 11b, c). Namely, this agreement strengthens the notion that rather endogenous or cell-to-cell transport of S1P is involved in controlling VE-cadherin mRNA expression levels, while exogenous S1P does not modulate its synthesis. As alluded to above, it is possible that exogenous S1P supplementation failed to affect changes in the VEGF-A expression at very low concentrations because S1PR3 may already be maximally activated rendering it unresponsive to S1P supplementation.

### Roles of S1PR3 signaling in mediating control of VEGF mRNA expression levels in HRMECs

**Fig. 12:**
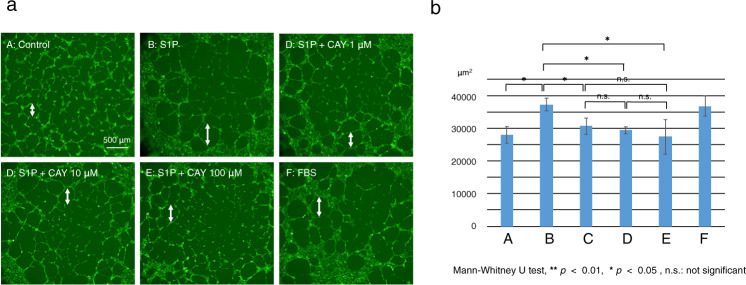
Roles of S1PR3 signaling in mediating angiogenic activity in HRMEC. **a** Coarser honeycomb structure formation (double arrow in each Frame) is indicative of increased HRMEC angiogenic activity in a culture containing S1P (B) as well as a positive control culture supplemented with 10% fetal bovine serum (FBS, F) had a coarser honeycomb structure as compared with the control culture (A) without any S1P supplementation. Such enhanced angiogenic activity of HRMECs by S1P administration was blocked in the presence of CAY10444 (C, D, E). Bar Scale, 500 μm. Frame **b** shows the averaged data and statistical analysis.

The extent of honeycomb formation under differential experimental conditions was used to characterize the involvement of the S1P/SPR3 signaling pathway axis in mediating control of HRMEC angiogenic activity [32] This was done based on monitoring, fluorescein stained cells under a microscope after 10 h of culture (Fig. 12a, b). In the S1P- supplemented culture as well as positive control culture containing 10% FBS, the honeycomb structure was coarser than in the control culture without any supplementation. In the presence of CAY10444, S1P-failed to enhance HRMEC angiogenic activity (Fig. 12a, b). Taken together, these results document that stimulation of the S1P/ SPR3 signaling pathway axis promotes neovascularization through upregulating VEGFA gene expression in HRMEC and HUVEC.

## Discussion

**Fig. 13:**
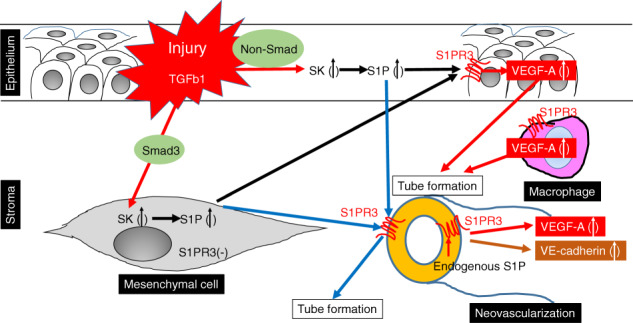
Proposed mechanism of induction of corneal neovascularization by TGFβ1/S1P/S1PR3 signaling cascade. Cauterization injury in the cornea upregulates TGFβ1, which promotes SPK1 expression in the corneal epithelium or stromal cells via either a Samd3-independent or Smad3 dependent mechanism, respectively. These cell lineages upregulate S1P expression as a consequence of increased SPK1 activity, which promotes generation of S1P. It is locally released and stimulates the epithelium to upregulate VEGF-A expression. Moreover, S1P promotes vessel structure formation by S1PR3- expressing vascular endothelial cells. Endogenous S1P in turn promotes VEGF-A upregulation.

We show here that activation by cauterization of the S1P/ S1PRP3 signal pathway axis contributes to corneal stroma neovascularization since this response was larger in the WT mice than in the S1PR3 knockout counterpart. This difference exists because S1PR3 is one of the five different candidate cognate receptor isoforms through which S1P-interacts to induce a host of different responses in different tissues. The functional association between S1P and S1PR3 in mediating this response was confirmed by showing that S1PR3 expression was upregulated by S1P in HRMECs and HUVECs as well as TKE2 corneal epithelial cell line. However the expression of this signaling pathway axis is cell type specific since there was no detectable S1PR3 immunostaining in situ keratocytes nor cultured ocular fibroblasts obtained from WT mice. S1P increases angiogenesis through interacting and stimulating S1PR3-linked signaling and in turn upregulating VEGF-A mRNA expression in the WT corneal stroma. Such rises stimulated endothelial cell proliferation based on increased CD31 immuno-labeling of endothelial cells that aligned themselves to form tube-like nascent leaky capillaries in WT corneas. The dependence of VEGF-A upregulation on S1PR3 expression and S1P induced increases in neovascularization was confirmed by showing that in the S1PR3 KO mice cauterization-induced smaller rises in VEGF-A gene expression and less neovascularization than in WT corneas. These results show that cauterization-induced increases in S1P expression mediate neovascularization through interacting with S1PR3 and in turn upregulating VEGFA gene expression. It has been shown that S1PR3 expression has a critical role in mediating neovascularization in non- ocular tissues. This is evident since systemic administration of CAY10444, an S1PR3 antagonist, suppressed expression of angiogenic growth factors and neovascularization of a neoplasm in a mouse ovarian cancer model [33]. Systemic inhibition of S1PR1-3 isoforms reportedly attenuates development of experimental liver fibrosis in association with neovascularization inhibition in mice [34, 35]. These results show that the regulatory role of this bioactive lipid in promoting neovascularization is not cell context dependent. It is evident that the VEGFA-induced increases in neovascularization are dependent on S1P interaction with S1PR3 since VEGFA expression levels and neovascularization were lower in S1PR3 KO mice than in their WT counterpart. S1P induced neovascularization through upregulating S1PR3 expression only in TKE2 corneal epithelial cells, but not in fibroblasts. In the TKE2 cells, S1PR3 expression was very evident whereas it was very faint in fibroblasts. Its low level of expression in fibroblasts and the failure of S1P to upregulate VEGF-A gene expression shows that VEGF-A induced neovascularization is dependent on functional S1PR3 expression. This agreement between the dependence of rises in VEGF-A expression on S1P- induced S1PR3 upregulation in WT and S1PR3 KO mice and TKE2 cells supports the notion that injury-induced angiogenesis is dependent on S1PR3 function. On the other hand, fibroblasts were unresponsive suggesting that the low levels of S1PR3 expression in mesenchymal cells are insufficient to adequately upregulate VEGF to support neovascularization. There are multiple factors that induce neovascularization through numerous different receptor-linked signaling cascades [36]. Nevertheless, this study strongly suggests that inhibition of neovascularization in a mouse cornea is at least in part, attributable to a decline in epithelial VEGF-A expression level subsequent to a parallel inhibition of the S1P/S1PR3 signaling pathway cascade in TKE2 corneal epithelial cells. Although the systemic circulation is the major source of systemic S1P it is also synthesized in various cell types and then exported into the systemic circulation [37]. We speculate that locally derived S1P from the cornea may be adequate to induce sufficient VEGF- A to stimulate neovascularization. It was reported that TGFβ1 upregulates SPK1, the major kinase involved in phosphorylating sphingosine to generate S1P [38]. Our results indicate that TGFβ1 upregulated SPK1 in both ocular fibroblasts and TKE2 corneal epithelial cells. However, this response occurred through Smad3 upregulation not only.in fibroblasts since SIS3 blocked it whereas it had no effect in TKE2 cells. Therefore, TGFβ upregulation has dual functions during wound healing that include both myofibroblast transdifferentiation which leads to scarring and also neovascularization. Both of these responses are mediated through SPK induced increases in S1P generation and stimulation of the S1PR3 linked signaling pathway and in turn VEGF-A upregulation in the corneal stroma. Another aspect of S1PR3 function involves its contribution in mediating chemoattractant behavior in macrophages [39, 40]. We found that loss of S1PR3 function in macrophages did not alter infiltration of either macrophages or neutrophils into a cauterized corneal wound. Nevertheless, the expression level of VEGF-A in KO macrophages was lower as compared with that in a WT counterpart. It is possible that less VEGF-A expression in a KO macrophage may contribute to the attenuation of neovascularization in a S1PR3KO mouse and slower wound healing due to less macrophage infiltration*.* Regarding the roles of S1PR3 in promoting vascular endothelial cells to undergo neovascularization, exogenous S1P did not alter the expression level of VEGF-A in both HRMECs and HUVECs. On the other hand, adding an S1PR3 antagonist, CAY10444, significantly downregulated VEGF-A mRNA expression. This finding suggests that endogenous S1P binds to S1PR3 without traversing through the extracellular space and modulates VEGF-A mRNA synthesis in the vascular endothelial cells through an autocrine mechanism. S1PR3 signaling is reportedly involved in upregulating VE- cadherin expression [16, 41, 42]. Another alternative accounting for why S1P failed to affect changes in the VEGF-A expression is that at very low concentrations of endogenous S1P, S1PR3 may already be maximally activated rendering it unresponsive to S1P supplementation. Our results also showed that as with VEGF-A exogenous S1P did not affect the expression level of VE-cadherin. These negative effects are consistent with our results showing that S1P mediates VE cadherin upregulation through increases in VEGF expression [30, 31]. While adding CAY10444 downregulated VE-cadherin mRNA expression. the endogenous S1P/S1PR3 signaling pathway mediating VE-cadherin expression might be also positively related to S1PR3-mediated neovascularization. S1P promoted honeycomb structure formation in HRMECs, on the other hand, CAY10444 suppressed this formation. This inhibitory effect confirms that the increase in angiogenic activity of S1P is mediated through S1PR3 activation in vascular endothelial cells. Meanwhile the results of gene expression analysis showed that S1P didn’t upregulate VEGF-A mRNA expression levels in HUVECs and HRMECs, but CAY10444 inhibited this response. It is possible that these disparate effects could be due to the fact that S1PR3 is already maximally activated at a very low concentrations of an endogenous S1P, and S1P may be unable to upregulate VEGF-A. In summary, Fig. 13 provides a diagrammatic representation of a model describing how the S1P/S1PR3 signaling pathway axis controls neovascularization Corneal cauterization initially upregulates TGFβ1 expression levels, which in turn stimulates SPK1 expression in the corneal epithelium or stromal fibroblasts via either a Smad3-independent or Smad3 dependent mechanism, respectively. This kinase in each of these different cell lineages phosphorylates sphingosine to generate S1P. S1P is then locally released in the tissue and in turn activates the epithelial S1PR3- linked cascade to upregulate VEGF-A expression. Moreover, S1P promotes vessel structure formation through interacting with and activating S1PR3-linked signaling in vascular endothelial cells. This signaling pathway induces rises in VEGF-A expression. Improved management of corneal wound healing outcome may be realized through modulating TGFβ1- S1P-S1PR3 signaling pathway activity and suppressing neovascularization in a clinical setting.
